# Evaluation of the Cytotoxic Behavior of Fungal Extracellular Synthesized Ag Nanoparticles Using Confocal Laser Scanning Microscope

**DOI:** 10.3390/ijms17030329

**Published:** 2016-03-03

**Authors:** Taher A. Salaheldin, Sherif M. Husseiny, Abdullah M. Al-Enizi, Ahmed Elzatahry, Alan H. Cowley

**Affiliations:** 1 Nanotechnology and Advanced Materials Central Lab, Agriculture Research Center, PO Box 588 Orman, Giza 12619, Egypt; t1salah@hotmail.com; 2 Faculty of Women for Art, Science & Education, Ain Shams University, PO Box 11757 Alkurba, Cairo 11341, Egypt; husseinymoussa@women.asu.edu.eg; 3 Department of Chemistry, College of Science, King Saud University, Riyadh 11451, Saudi Arabia; amenizi@ksu.edu.sa; 4 Materials Science and Technology Program, College of Arts and Sciences, Qatar University, PO Box 2713, Doha, Qatar; 5 Department of Chemistry, University of Texas at Austin, Austin, TX 78712, USA; acowley@cm.utexas.edu

**Keywords:** silver nanoparticles, cytotoxicity, breast cancer, confocal laser scanning microcopy

## Abstract

Silver nanoparticles have been synthesized by subjecting a reaction medium to a *Fusarium oxysporum* biomass at 28 °C for 96 h. The biosynthesized Ag nanoparticles were characterized on the basis of their anticipated peak at 405 nm using UV-Vis-NIR spectroscopy. Structural confirmation was evident from the characteristic X-ray diffraction (XRD) pattern, high-resolution transmission electron Microscopy (HRTEM) and the particle size analyzer. The Ag nanoparticles were of dimension 40 ± 5 nm and spherical in shape. The study mainly focused on using the confocal laser scanning microscope (CLSM) to examine the cytotoxic activities of fungal synthesized Ag nanoparticles on a human breast carcinoma cell line MCF7 cell, which featured remarkable vacuolation, thus indicating a potent cytotoxic activity.

## 1. Introduction

Nanotechnology continues to attract significant attention due to its impact in many currently important areas such as energy, medicine, electronics and the aerospace industry. As might be anticipated, this field has been growing very rapidly on a worldwide basis over the past decade. Nanoparticles that possess one or more dimensions of the order of 100 nm or less continue to attract significant attention due to their unique properties in the realms of chemistry, optics, electronics and magnetism. As a consequence, there is an ever-increasing interest in the synthesis of such compounds [1,2].

Nanoparticles have been synthesized by a variety of physical and chemical processes. Unfortunately, however, some of these chemical methods cannot avoid the use of toxic chemicals that are needed for the synthesis process. Given the foregoing problem, there is an urgent need for the development of a more green process that will serve as an alternative to the current chemical and physical methods [3].

The use of eukaryotic organisms such as fungi offers considerable promise for large-scale metal nanoparticle production since the enzymes that are secreted by the fungi represent an essential ingredient for the biosynthesis of metal nanoparticles [4,5,6]. Several fungi such as *Verticillium* and *Fusarium oxysporum* have been reported to be useful for the synthesis of metal nanoparticles [5,7,8].

Out of all the metals with antimicrobial properties, silver has the strongest antibacterial action and the least toxicity. Silver is therefore particularly useful for the treatment of mammalian tissues where it acts as a potent antiseptic agent [9]. Moreover, in either its metallic or ionic form, silver exhibits cytotoxicity against microorganisms and is therefore particularly useful as an antimicrobial agent [10,11,12].

Silver nanoparticles (Ag NPs) has attracted high interest due to their unique and excellent properties in addition to its therapeutic potential for the treatment of a variety of diseases that includes retinal neovascularization [13,14] and acquired immunodeficiency syndrome due to human immunodeficiency virus (HIV) [15,16]. More recently, the antitumor effect of Ag NPs has been reported to be effective against a variety of cancerous cell lines [17,18,19]. Recently, we reported synthesis, size control optimization of Ag nanoparticles using fungus *Fusarium oxysporum* and their antimicrobial and antitumor activities [20].

In the present work, confocal laser scanning microscopy has been used to study the efficiencies of the Ag NPs that were synthesized extracellular by treatment with the fungus *Fusarium oxysporum* (*F. oxysporum*) and followed by a WST1 cytotoxicity measurement.

## 2. Results and Discussion

### 2.1. Extracellular Biosynthesis of Fungal Ag NPs

**Figure 1:**
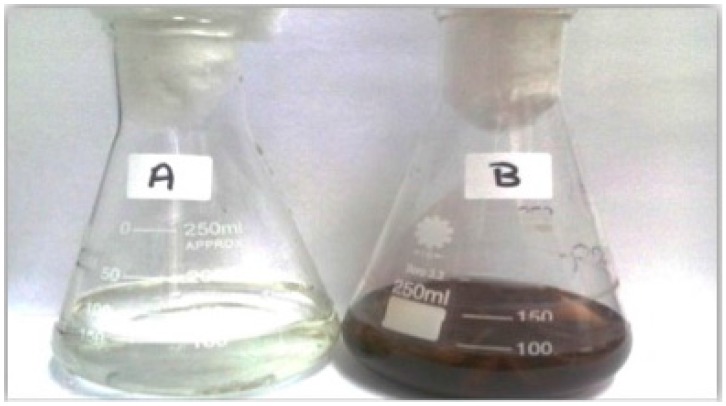
Progress of the biosynthesis process of Ag nanoparticles by *Fusarium oxysporum* filtrate: (**A**) at zero time after addition of 10^−3^ M AgNO_3_ and (**B**) after 72 h.

The biosynthesis of metal nanoparticles using microorganisms is a well-known technique that has reported in several useful applications [2,3]. The present study used local *Fusarium oxysporum* for synthesis of silver nanoparticle at optimal conditions*.* In accordance to previous reports, upon mixing the addition of silver nitrate to a filtered cell-free culture, a yellowish brown color appeared as a result of Ag nanoparticles formation and its intensity increased with the incubation time [20,21,22]. Figure 1 represent the color change as a visual indicator of the progress of the biosynthesis process at zero time (A: colorless) and after 72 h (B: yellowish-brown). The appearance of a dark-brown color in the fungal cell filtrate is due to excitation of surface plasmon after treatment with silver nitrate and is furthermore indicative of the synthesis of Silver Nanoparticles, SNPs, exhibits strong absorption in the visible range due to the local surface plasmon resonance [21,22,23].

### 2.2. Characterization of Fungal Ag NPs

**Figure 2:**
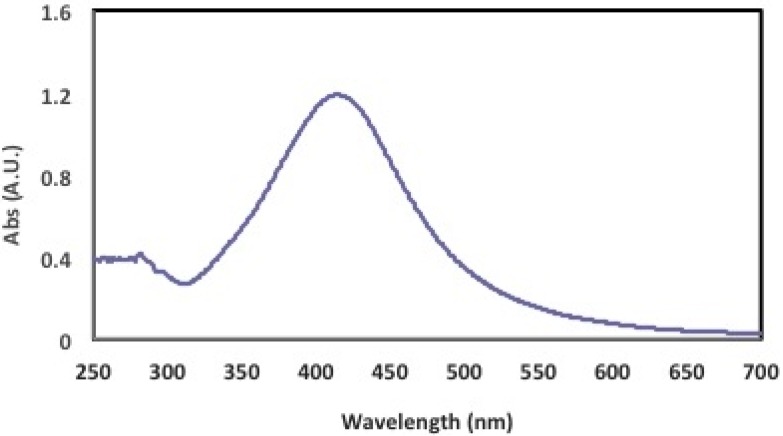
Spectrophotometric absorption peak at 413 nm of AgNPs synthesized by *Fusarium oxysporum*.

UV-Vis spectra of prepared Ag NPs sample is displayed in Figure 2. During the synthesis of the SNPs, an absorption spectrum with a sharp peak at 413 nm became apparent, which corresponded to the plasmonic absorption band of the silver nanoparticles. Furthermore, the presence of a single peak was indicative of the synthesis of spherical nanoparticles. It is well known that there is a very close relationship between the UV-Vis absorbance spectrum and the size and shape of SNPs. With an increase in particle size, the optical absorption spectra of metal nanoparticles that are dominated by surface plasmon resonances (SPR) are shifted toward longer wavelengths (redshift) [24].

**Figure 3:**
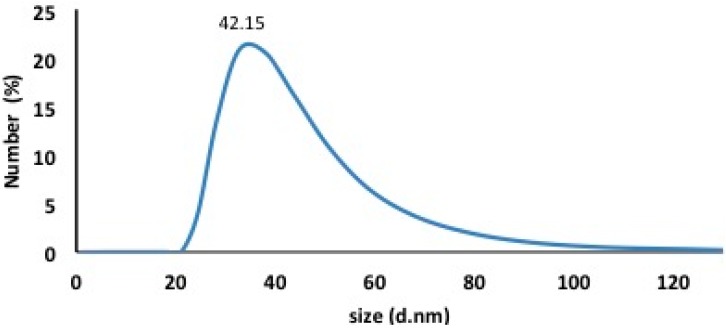
Particle size distribution shows 42.15 nm mean particle size.

**Figure 4:**
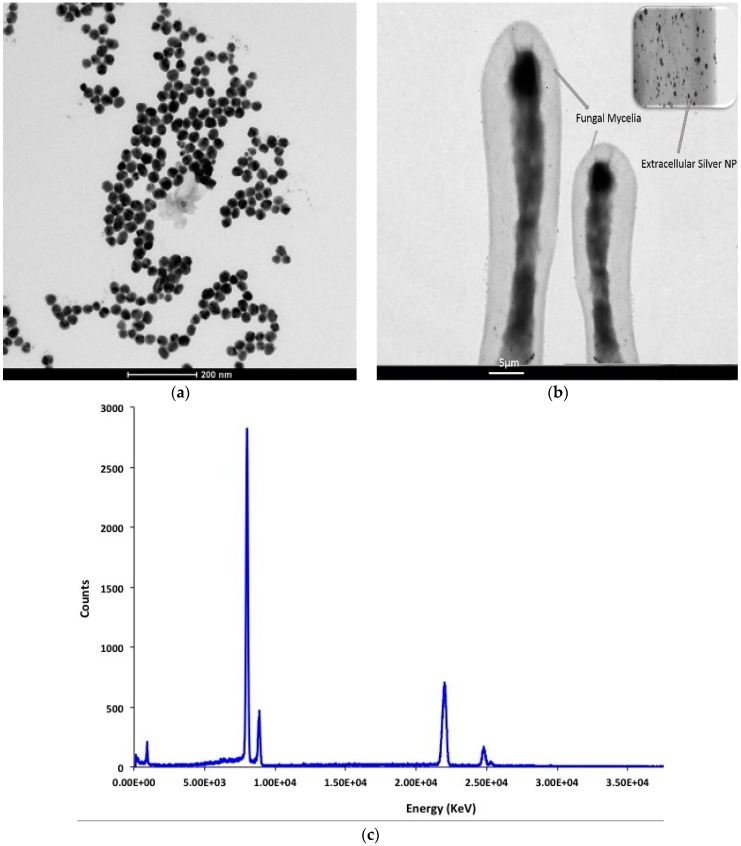
Transmission Electron Microscopy (TEM) images of silver nanoparticles synthesized by *F. oxysporum* show: (**a**) spherical particles with mean size distribution 40 ± 5 nm; (**b**) extracellular synthesis by fungal mycellia; and (**c**) EDX results,

The use of Dynamic Light Scattering (DLS) techniques permitted the measurement of the size distribution of the newly synthesized silver nanoparticles. The average size of the silver particles was about 42.15 ± 3.5 nm, as shown in Figure 3. Moreover, the TEM imaging was performed to determine the extracellular synthesis of silver nanoparticles by fungal mycelia in addition to the morphologies and shapes of nanoparticles. Figure 4 shows the formation of tiny silver nanoparticles with average size 40 ± 5 nm on the surface of the fungal mycelia, confirming the extracellular approach for synthesis and EDX measurements for the High Resolution Transmission Electron Microscope (HRTEM) image confirm the phase formation of silver nanoparticles. In addition, the silver nanoparticles were of approximately spherical shape and good quality and uniform distribution (monodispersed) without significant agglomeration. These results are in accordance with those described in [23].

**Figure 5:**
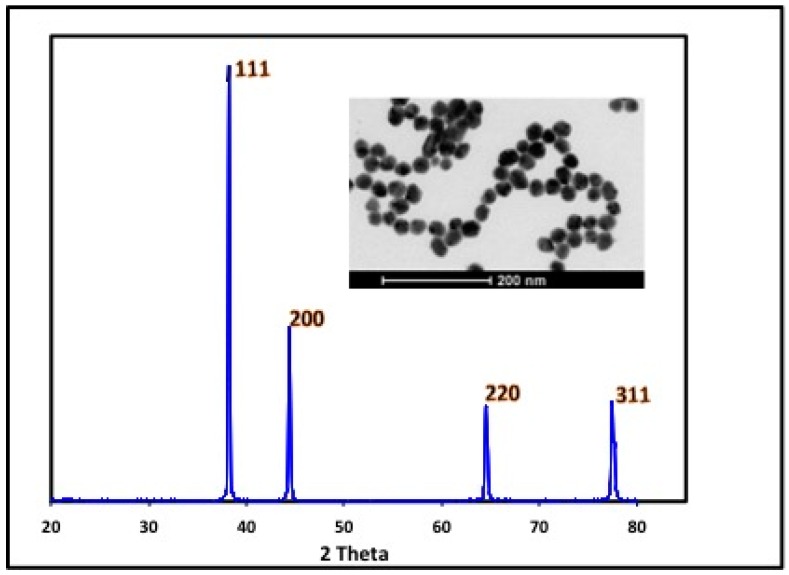
XRD pattern of as-synthesized silver nanoparticles produced by *F. oxysporum.*

The formation of silver nanoparticles was also confirmed by the presence of an X-ray diffraction phase pattern with narrow peaks, which is indicative of the crystalline nature of the Ag NPs. Furthermore, intense XRD peaks were observed that correspond to the (111), (200), (220) and (311) planes at 2θ angles of 38.11°, 44.12°, 64.24°, and 77.52°, respectively. Additionally, these results were in good agreement with those of the unit cell anticipated for a face-centered cubic (fcc) system (Figure 5) [4]. In general, the breadth of a specific phase of a material is directly proportional to the mean crystallite size of that particular material and the presence of broader peaks indicates that the crystallite size is small [25]. Taken collectively, the foregoing measurements confirm the ability of *Fusarium oxysporum* to reduce silver nitrate thereby forming the silver nanoparticles under controlled experimental conditions that are in accord with the literature values [23,26].

### 2.3. Cytotoxic Activity of Fungus Ag NPs

**Figure 6:**
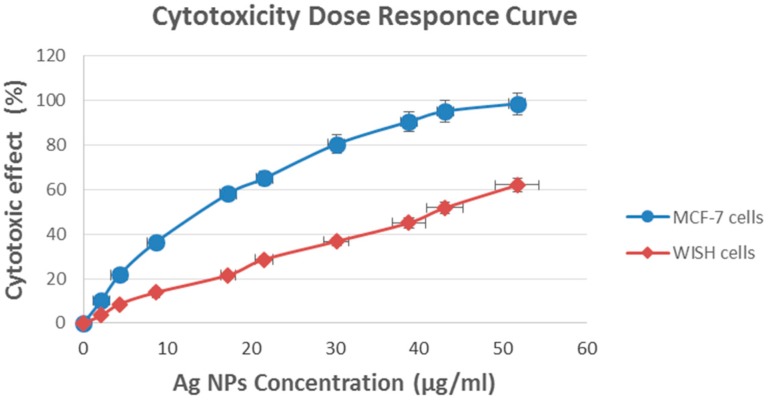
Cytotoxic effect of AgNPs synthesized by *Fusarium oxysporum* against MCF-7 and normal WISH cell lines

*In vitro* model of human breast carcinoma cells (MCF-7) and normal WISH cells (Human normal fibroblast cell) were used to study the cytotoxic effect of the aqueous suspension of the synthesized silver nanoparticles after filtration through a 0.22 μm syringe driven filter unit. The cells were cultured in DMEM, and maintained at 37 °C and humidified with 5% CO_2_. In the case of sub-culturing, the monolayer cells were harvested after treatment with trypsin/EDTA at 37 °C. The WST-1 Cellular proliferation assay was used to evaluate the cytotoxicological activities of a variety of concentrations of the Ag NPs that were being tested (0, 2.2, 4.3, 8.6, 17.3, 21.6, 30.2, 38.8, 43.2, and 51.8 µg/mL) against a human breast carcinoma cell line (MCF-7) and the normal WISH cell line for comparison. The selected doses were added to the cell monolayers in triplicate wells and the cytotoxicity of each individual dose was tested using a standard WST-1 assay for the rapid and sensitive quantification of cell proliferation and viability [20]. Furthermore, the WST-1 assay results revealed that the fungal Ag NPs that were synthesized using the above procedures have a promising cytotoxic activity against the human breast carcinoma cell line (MCF-7) compared to the normal WISH cells. The significant decrease in the mitochondrial dehydrogenase activity as a function of the growth rate of the tumor cells is attributable to cleavage of the tetrazolium salt WST-1 to formazan by cellular mitochondrial dehydrogenases. Moreover, it was clearly apparent that the cytotoxic effect was concentration dependent. The cytotoxic effect of the tested compounds in response to the concentrations gradient is illustrated in Table 1 and Figure 6.

IC_50_, the dose required to kill 50% of the cultured cell population, can be estimated form the dose–response curve plotted using the WST-1 assay results where the cytotoxic activity can be expressed as the mean IC_50_ of three independent experiments [27]. IC_50_ was directly estimated from actually experiment data and found to be 14 µg/mL for MCF-7 cells and 42 µg/mL for WISH cells. The small IC_50_ value the as-prepared Ag NPs for MCF-7 cell line compared to the normal WISH cell line exhibited impressive efficiencies as a cytotoxic drug, which are in accord with literature values [28,29,30]. The higher cytotoxicity for malignant cells (MCF-7) compared to healthy normal cells (WISH) might be attributable to the high proliferation and oxidative stress in the malignant cells. It is worth mentioning that the cytotoxic effects of Ag nanoparticles against normal or abnormal cell line are mostly well studied and previously reported [31,32].

### 2.4. Confocal Laser Microscopic Mode of Action

**Figure 7:**
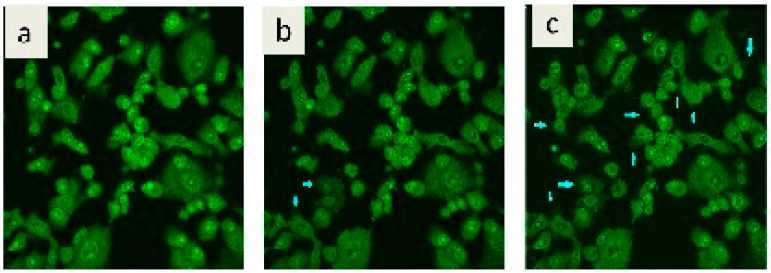

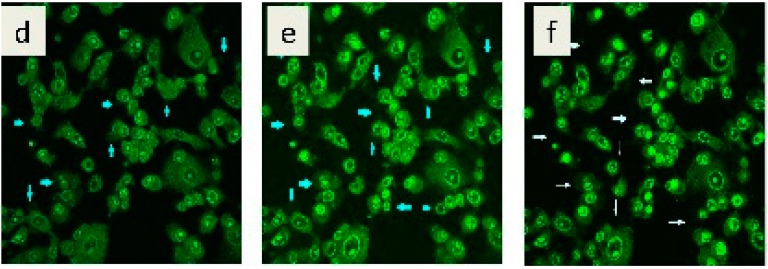
Confocal laser scanning microscopic imaging indicating the development of vacuoles formation as a function of incubation time: (**a**) at zero time, no vacuole formed; (**b**) after 2 h, start vacuoles formation (arrows); (**c**) after 4 h, increase vacuolization rate (arrows); (**d**) after 6 h, increased vacuoles size (arrows); (**e**) after 8 h, intact vacuoles outside cells (arrows); and (**f**) after 10 h, over vacuolization everywhere from all cells sides (arrows). Magnification 40×.

It is well known that WST-1 assay results reveal a significant decrease in the mitochondrial dehydrogenase activity as a function of the growth rate of the tumor cells. However, the foregoing approach did not explain the cytotoxic mode of action of the tested fungal Ag NPs [1]. Confocal laser scanning microscopic (CLSM) imaging technique can add valuable knowledge about the behavior of the cells under Ag nanoparticles stress. Human breast carcinoma cell line (MCF-7), stained with acridine orange dye and treated with 20 µg/mL Ag NPs, revealed remarkable intracellular vacuolation, which is indicative of potent cytotoxic activity [33,34,35]. The rate of vacuole formation is dependent upon the amount of stress that is placed on the cells due to the Ag NPs. As a consequence, tested cells attempt to regenerate themselves via a vacuolization process that is clearly illustrated in both Figure 7 and supplementary Video 1. It is well known that the appearance of vacuole formation is a sign of apoptosis due to the highly toxic effect of the Ag NPs. This conclusion was confirmed by means of a WST-1 proliferation assay 24 h after incubation.

## 3. Materials and Methods

### 3.1. Microorganisms

The low cost of the fungal plant pathogen strain *F. oxysporum* *f.sp. lycopersici* EMCC 632 was obtained from Microbiological Resources Centre (MIRCEN, Cairo), Egypt. The fungus was maintained on potato dextrose agar slants at 28 °C and sub-cultured from time to time in order to regulate its viability. This medium consisted of an infusion of potatoes (200 g), dextrose (20 g), and 1 liter of distilled water. The mixture was autoclaved at a pressure of 1.5 atmospheres for 20 min.

### 3.2. Biomass Production

The strain was grown aerobically to produce the biomass in 250 mL capacity Erlenmeyer flasks, each containing 100 mL of sterile potato dextrose broth. The flasks were inoculated with a spore suspension of *F. oxysporum* *f.sp. lycopersici* and incubated at 28 °C for 7 days. After incubation, the biomass was separated from the medium by filtration through Whatman filter paper No. 1 and washed three times with Milli-Q-deionized water to remove any medium components from the biomass.

### 3.3. Biosynthesis of Silver Nanoparticles

In a typical biosynthesis of Ag Nps, 10 g of *F. oxysporum* *f.sp*. *lycopersici* biomass was transferred to a flask that contained 100 mL of deionized water. Each flask was attached to a rotary shaker operating at 180 rpm at 28 ± 2 °C for 72 h. Following this, the biomass was separated by filtration and the aqueous filtrate was used for the biosynthesis of the nanoparticles. In the next step, silver nitrate was added to the aqueous mycelial free filtrate in a 250 mL flask until a final concentration of 10^−3^ M was achieved. The latter solution was maintained at 28 °C for 96 h. Simultaneously, controls of the aqueous filtrate and the silver nitrate solution were made using the same conditions [2,3].

### 3.4. Characterization of Synthesized Silver Nanoparticles

The reaction media of fungal suspension containing Ag nanoparticles was filtered through a 0.22 μm syringe filter to remove any fungal residue from the supernatant. Golden yellow clear solution of Ag nanoparticles was obtained for characterization. In order to examine the existence of non-reacted free Ag^+^ ions in the colloidal solution of silver nanoparticles, sodium chloride salt solution was added to the synthesized Ag nanoparticles. The solution turned turbid if in presence of non-reacted Ag free ions. Formation of totally clear golden yellow solution reveals that all the free Ag ions were reacted forming Ag nanoparticles. Characterization of the Ag nanoparticles was carried out using different techniques. Absorption spectrum was recorded on a Varian, Carey 5000 spectrophotometer (Agilent Technologies, Santa Clara, CA, USA). The size distribution measurements of the silver nanoparticles were carried out by a dynamic light scattering (DLS) technique (Malvern Zeta Sizer-Nano series, Malvern, Worcestershire, UK) and the high resolution Transmission Electron Microscopic imaging was performed by FEI (Eindhoven, The Netherlands), Tecnai G2 and X-ray diffraction for phase analysis by PanAlytical, X’Pert Pro (Almelo, The Netherlands).

### 3.5. Cytotoxic Activity

#### 3.5.1. MCF-7 Cell Culture

The human breast carcinoma cell line (MCF-7) was cultured and used to evaluate the cytotoxic effects of the tested extracts at the Nanotechnology & Advanced Materials Central Laboratory, Cairo, Egypt. A routine MCF-7 cell culture protocol was followed. Ready made cultured media, DMEM, (Dulbecco’s Modified Eagle’s Medium, Lonza, Waverley, Australia) was used for cellular growth and 250 ng/mL of amphotericin B and 100 units/mL of streptomycin sulfate. The culture was maintained at 37 °C and humidified with 5% CO_2_ for sub-culturing. The monolayer cells were harvested after treatment of trypsin/EDTA at 37 °C.

#### 3.5.2. WST-1 Assay

The cytotoxicological activities of various concentrations of the Ag NPs being tested (0, 2.2, 4.3, 8.6, 17.3, 21.6, 30.2, 38.8, 43.2, and 51.8 µg/mL) were evaluated using cultures of MCF-7 human breast adenocarcinoma cells as an *in vitro* model of breast cancer and to compare the results with human normal fibroblasts cells (WISH cells). For this purpose, selected doses were added to the cell monolayer in triplicate wells and their cytotoxicities were tested using standard WST-1(4-[3-(4-iodophenyl)-2-(4-nitrophenyl)-2*H*-5-tetrazolio]-1,3-benzene disulfonate) assays as a rapid and sensitive quantification of cell proliferation and viability [36].

#### 3.5.3. Confocal Laser Scanning Microscopy (CLSM)

Confocal laser scanning microscopic (Carrl Zeiss CLSM 710, Jena, Germany) was used to evaluate the cytotoxicity action of Ag NPs by imaging of the MCF-7 treated cell lines at an IC_50_ concentration of the synthesized Ag NPs. Samples were prepared according to the literature [37]. In brief, the MCF-7 cells were placed in 96- Multiwall plates (approximately 10^4^ cells/well) for 24 h prior to treatment with the tested compound, thereby allowing attachment of each individual cell to the glass base of the plate. Selected concentrations of the cells being tested were added to the cell monolayer in triplicate wells of individual doses. The monolayer cells were incubated with the compounds for 24 h at 37 °C and in an atmosphere of 5% CO_2_. After 24 h, the cells were stained by Acridine Orange dye obtained from Sigma Aldrich (Cairo, Egypt). After a delay of five minutes, microscopic examination of the cells was carried out using the excitation lines at 633 nm and single channel detection.

## 4. Conclusions

The production of SNPs using aqueous extracts of the fungus *F. oxysporum* is a promising candidate for the low-cost and environmentally friendly production of stable and uniformly sized SNPs with anticancer activities. Furthermore, structural confirmation was provided by the characteristic XRD pattern. The HRTEM and particle size distribution revealed that the Ag NPs were of dimension 40 ± 5 nm and spherical in shape. The newly synthesized Ag NPs were characterized by the appearance of the characteristic peak at 413 nm using UV-Visible-NIR spectroscopy. The results obtained in the present work open several new avenues for further study, such as the purification and biochemical characterization of the reductase produced by *F. oxysporum* and the development of an alternative Ag NPs formulation that reduces the toxicity of silver. Furthermore, CLSM is an important tool for enhancement of the localization, visualization and penetration of Ag NPs synthesized by *Fusarium oxysporium* in the MCF7 cell line. CLSM imaging technique can help in exploring the mode of action of tested nanomaterial and the cellular behavior upon treatment. Overall, the present report describes a cost effective, single step and eco-friendly synthesis of Ag NPs that could find more safe applications in drug delivery and cancer diagnosis and treatment.

## Figures and Tables

**Table 1:** Percentage of cytotoxic effect of fungal Ag NPs against MCF-7 and normal cell lines.

Concentration (µg/mL)	Cytotoxic Effect (%)	
	MCF-7 Cells	Normal WISH Cells
0	0	0
2.2	10.2 ± 2.7	3.5 ± 2.3
4.3	21.7 ± 2.4	8.6 ± 2.1
8.6	36.4 ± 2.1	13.8 ± 2.0
17.3	58.1 ± 1.8	21.6 ± 1.6
21.6	65.1 ± 1.5	28.6 ± 1.2
30.2	80.5 ± 1.0	36.8 ± 1.3
38.8	90.6 ± 0.9	45.1 ± 0.8
43.2	95.2 ± 0.6	51.8 ± 1.3
51.8	98.7 ± 0.7	62.1 ± 2.0

## Supplementary Materials

Supplementary materials can be found at http://www.mdpi.com/1422-0067/17/3/329/s1.
